# Cognitive Neurorehabilitation in Acquired Neurological Brain Injury

**DOI:** 10.1155/2019/8241951

**Published:** 2019-11-05

**Authors:** Lambros Messinis, Mary H. Kosmidis, Grigorios Nasios, Efthymios Dardiotis, Theodore Tsaousides

**Affiliations:** ^1^Neuropsychology Section, Departments of Neurology and Psychiatry, University Hospital of Patras and University of Patras Medical School, 26504 Patras, Greece; ^2^Lab of Cognitive Neuroscience, School of Psychology, Aristotle University of Thessaloniki, Thessaloniki, Greece; ^3^Department of Speech and Language Therapy, School of Health Sciences, University of Ioannina, Ioannina, Greece; ^4^Department of Neurology, University of Thessaly Medical School, Larissa, Greece; ^5^Icahn School of Medicine at Mount Sinai, New York City, USA

Cognitive impairment is frequent after acquired neurological brain injury (ANBI) and may reduce the ability of a person to adequately process and interpret information, leading to abnormal behaviors, mood disturbances, and dysfunctional adaptive strategy formation. These disabilities may cause profound limitations, affecting daily functional capacity, vocational activities, and socialization [1].

Although a wealth of empirical research has investigated the behavioral and cognitive consequences associated with acquired brain injuries (ABI), relatively less rigorous research has been devoted to investigating their rehabilitation. The main objective of any rehabilitation intervention is to maximize functional recovery and independence, reinstate employment, achieve functional productivity, and improve overall quality of life. Cognitive neurorehabilitation or cognitive rehabilitation (CR) is usually the treatment of choice for impaired cognition and co-occurring behavioral disturbances. Cicerone et al. [2] define CR as “a systematic, functionally oriented service of therapeutic activities that is based on assessment and understanding the patients' brain–behavioral deficits.”

Cognitive rehabilitation is recognized as a standard component of rehabilitation programs for patients with ABI in many national clinical guidelines (e.g., European Federation of Neurological Sciences) [3]. It is a therapeutic approach aiming to improve cognitive functioning, such as attention, learning and memory, problem solving abilities, and executive function, but also affect and expression [2, 4]. It includes an assembly of methods which attempt to retrain (restore) lost functions or previously established behavioral patterns, with an emphasis in training activities within everyday contexts. Alternatively, compensatory (adaptive) strategies may be taught and applied by the patient or others in order to circumvent their limitations [5]. Addressing the psychological impact and consequences of the brain injury and establishing individual variability patterns are also considered important factors in achieving optimal functional capacity.

In 2002, the National Academy of Neuropsychology (NAN) published an official statement on CR that supports empirically and rationally based CR techniques that have been designed to improve the quality of life and functional outcomes for individuals with ABI (NAN 2002). The American Congress of Rehabilitation Medicine (ACRM), Brain Injury Interdisciplinary Special Interest Group (BI-ISIG), after conducting a systematic review on the impact of cognitive rehabilitation for persons with traumatic brain injury (TBI) or stroke, found a differential benefit favoring CR in almost 80% of all treatment comparisons [2, 6, 7]. Based on these findings, recommendations for clinical practice were formulated accordingly.

CR interventions can usually be applied at all stages of post-injury recovery and in different settings (e.g., inpatient, outpatient, and domestic). Additionally, they can be administered with different modalities (e.g., individual, family, and group) and by healthcare professionals trained in different clinical disciplines (e.g., speech-language therapists-pathologists, clinical neuropsychologists, and occupational therapists) Turner-Stokes et al. [8] investigated multidisciplinary rehabilitation for ABI in adults of working age in a Cochrane review and reported that the context of multidisciplinary rehabilitation appears to positively influence therapeutic outcomes. “Moreover, they found strong evidence” supporting comprehensive CR in a therapeutic environment that involves a peer group of patients.

However, despite previous reports on the positive impact of CR in ABI, the benefits reported are mostly short term and do not generate to everyday contexts. Moreover, systematic reviews generally conclude that the efficacy of CR in ABI, whether of a progressive or nonprogressive nature, is uncertain. In this respect, a systematic review by Koehler et al. [9], found limited, and in some cases, modest evidence that CR is effective for treating some deficits related to traumatic brain injury (TBI), including attention, executive function, social communication, and memory. In 2013, Chung et al. [10] found insufficient evidence that CR interventions were helpful for individuals with executive dysfunction or other secondary outcome measures. More recently, a Cochrane review by Kumar et al. [4] noted that “there is insufficient evidence to support the role of CR when compared to no intervention or conventional rehabilitation in improving return to work, independence in activities of daily living, community integration or quality of life in adults with TBI.”

In the most recent and updated systematic review [11] that investigated the relevant clinical literature on the efficacy of CR interventions in persons with TBI from 2009 to 2014, the authors who were members of the Cognitive Rehabilitation Task Force (CRTF) of the ACRM concluded that the evidence obtained supports Practice Standards for (i) attention deficits after TBI or stroke, (ii) visual scanning for neglect after right hemisphere stroke, (iii) compensatory strategies for mild memory deficits, (iv) language deficits after left hemisphere stroke, (v) social-communication deficits after TBI, (vi) metacognitive strategy training for deficits in executive functioning, and (vii) comprehensive-holistic neuropsychological rehabilitation to reduce cognitive and functional disability after TBI or stroke. Although the findings of this recent systematic review provide sufficient evidence to formulate Practice Standards for various CR interventions, the authors note that there is limited evidence that the benefits obtained translate into long-term meaningful changes in patients' everyday functioning capacity. Moreover, specific patient characteristics or models of treatment delivery that impact intervention success are not clearly evident. In this sense, further high-quality research that is directed towards identifying specific patient characteristics (e.g., psychological insight, cognitive reserve, and psychiatric comorbidity) and treatment delivery variables (i.e., intensity and frequency) is an important requirement.

On this background, this special issue entails a series of cutting-edge articles that provide innovative research findings and recent information and advances in the rehabilitation mainly of adults with cognitive, behavioral, or emotional difficulties or disorders resulting from stroke, TBI, or other ABI. We have included original research articles, systematic and Cochrane reviews, and meta-analytic explorations.

Stalnacke et al. from Sweden in an intriguing article investigated through a prospective cohort study the clinical course of disability, cognitive, and emotional impairments in patients aged 18-65 with severe TBI (s-TBI), from 3 months up to 7 years post TBI. The findings of this study are important as most studies of s-TBI focus on the acute phase and then possibly a short follow-up period. In this respect, information regarding the long-term cognitive, emotional, and behavioral outcomes of this heterogeneous clinical population are lacking in the literature. The authors reported that speech and language functions, orientation, attention/concentration, visuospatial and visual problem solving, memory, affect, and awareness as measured by the Barrow Neurological Institute Screen for Higher Cerebral Functions (BNIS) [12] improved significantly from 3 months to 1 year, but not from 1 year to 7 years post TBI. Furthermore, disability status as measured by the 8-category Glasgow Outcome Scale Extended (GOSE) [13] also improved significantly from 3 months to 1 year, but not from 1 year to 7 years post TBI. The findings indicate that s-TBI patients appear to improve during the first year post trauma, and then remain relatively stable at least until the 7^th^ year post trauma. These results highlight the necessity of initially screening for cognitive functions post TBI and then following up with additional assessments over time in order to provide reliable rehabilitation interventions in this population.

De Luca et al. from Italy provide important new data in their compelling article, supporting the ecological validity and efficacy of CR interventions utilizing virtual reality (VR) over traditional CR in patients with TBI. The VR program utilized in this study, according to the authors, offers interactive virtual scenarios and audiovisual stimuli through movement, creating a total sensory involvement that facilitates rehabilitation of attention, visual-spatial, and executive skills. This is carried out with the help of a therapist. Findings of this study showed that cognitive flexibility, shifting skills (i.e., executive functions), and selective attention improved only in the group that received the VR intervention, three sessions a week for 8 weeks compared to the group that received traditional CR. These findings may have implications for the formulation of Practice Standards for CR interventions, possibly directed towards the utilization of more ecologically directed VR-based programs. However, additional evidence from large-scale studies applying VR programs will be necessary in order to investigate whether the benefits obtained translate into long-term meaningful changes in these patients' everyday functioning abilities.

Constantinidou from Cyprus provides us with an appealing article investigating the effects of hierarchical cognitive training using the categorization program (CP) in patients with TBI, noninjured young adults (18-50), and adults over 60 years old. According to the author, the CP is a rigorous systematic, hierarchical, eight-level program designed as a restorative CR program in adults with ABI. It addresses two distinct areas of human categorization, i.e., passive object recognition and new category learning. Previous research supports its efficacy for adults with ABI who exhibit categorization deficits [14, 15]. Findings of the present research indicated significant improvement in categorization performance in all treated groups, with younger participants, whether healthy or with TBI, demonstrating greater performance gains. Interestingly, a subgroup of older trained adults maintained their positive performance gains for at least four months post intervention. These interesting findings, according to the author, may have implications for how to improve adult cognitive learning and formulate future CR interventions.

Corti et al. from Italy provide an intriguing Systematic Review and Meta-Analytic Exploration on Remote Technology-Based Training programs (RTBP) for children with acquired brain injury. The article provides us with the most recent cognitive and behavioral features and advances related to these types of CR interventions in the child population. The impact of an ABI during childhood is significant, especially with regard to core cognitive functions, behavioral disturbance, social difficulties, and academic performance [16]. Addressing the cognitive and behavioral consequences of ABI in this population becomes a major priority, and RTBP, according to the review and meta-analysis, represent a promising rehabilitation endeavor in this respect. The authors conclude, however, that further rigorous research is required to identify which training characteristics of RTBP and population subgroups may benefit more from such interventions.

Gomez-Gastasoro et al. from Spain focus their interesting article on reviewing the efficacy of an integrative CR program (REHACOP) that provides an integrated bottom-up and top-down approach to rehabilitation, hierarchically organized on an increasing level of difficulty. They stipulate that the application of the program on different neurological populations with cognitive impairment and deficits in social cognition (i.e., multiple sclerosis and Parkinson's disease) and in patients with schizophrenia positively impacted cognition and daily living tasks. The magnitude of improvement ranged from medium to high across the various samples.

Ardilla and. Roselli, from the USA, provided us with an alluring review article on acalculia (or acquired dyscalculia), which according to the authors, is a disturbance in understanding the numerical system associated with the loss of the ability to perform arithmetical operations. The literature related to the rehabilitation of acalculia is relatively limited. Moreover, calculation or numerical difficulties are not commonly evaluated during neuropsychological assessments, despite the fact that acalculia can represent a fundamental acquired cognitive disturbance (primary acalculia). In this respect, this article may provide useful information in order to assist clinicians in rehabilitation settings to address an unmet need.

The compelling review article by Nasios et al. from Greece shifts our attention to the neural networks subserving language and extends from the era of autopsy-driven research with the Broca-Wernicke-Lichtheim-Geschwind classical model to the new model of the functional neuranatomy of language, i.e., the dual stream model. Advances and new insights of language studies in the aging brain and also language in young/developing brains are also critically reviewed. The article further comments on the linkage between language and cognition especially in the elderly, as well as in poststroke aphasia, and the reorganization of language networks in order to provide improved neurorehabilitation strategies for people suffering from neurogenic communication disorders.

Finally, the stirring review article by Patrikelis et al. from Greece explored the current knowledge and assessed the evidence linking secondary (acquired) alexithymia to aberrant humor processing, via their neurobiological mechanisms. Additionally, a possible common neuropathological substrate between secondary alexithymia and deficits in humor appreciation was investigated by drawing on neurophysiologic and neuroradiological evidence, as well as on a recent and unique single-case study which showed the cooccurrence of secondary alexithymia and deficit in humor appreciation. The authors conclude that increased awareness on this topic may assist neurosurgeons when accessing emotion-relevant structures, and for neuropsychologists when formulating evidence-based neurorehabilitative interventions.

From the reports discussed previously and the contributing articles in this special issue, it becomes apparent that significant progress has been achieved in the cognitive rehabilitation of ANBI. Although the efficacy of CR to translate into meaningful long-term changes in ANBI patients' everyday functioning abilities remains uncertain, several important clinical trends are apparent, that require large, high-quality trials, with ecologically valid interventions in order to elucidate patients' everyday functioning capacity. We suggest careful research planning directed towards difficulties in daily life must form the basis for further studies on the efficacy and effectiveness of CR treatment interventions in these diverse clinical population groups.

## References

[1] Whyte E., Skidmore E., Aizenstein H., Ricker J., Butters M. (2011). Cognitive impairment in acquired brain injury: a predictor of rehabilitation outcomes and an opportunity for novel interventions. *PMR*.

[2] Cicerone K. D., Dahlberg C., Kalmar K. (2000). Evidence-based cognitive rehabilitation: recommendations for clinical practice. *Archives of Physical Medicine and Rehabilitation*.

[3] Members of the Task Force on Cognitive Rehabilitation, Cappa S. F., Benke T. (2005). EFNS guidelines on cognitive rehabilitation: report of an EFNS task force. *European Journal of Neurology*.

[4] Kumar K. S., Samuelkamaleshkumar S., Viswanathan A., Macaden A. S., Cochrane Work Group (2017). Cognitive rehabilitation for adults with traumatic brain injury to improve occupational outcomes. *Cochrane Database of Systematic Reviews*.

[5] Koehler R., Wilhelm E. E., Shoulson I. (2011). *Cognitive Rehabilitation Therapy for Traumatic Brain Injury: Evaluating the Evidence*.

[6] Cicerone K. D., Dahlberg C., Malec J. F. (2005). Evidence-based cognitive rehabilitation: updated review of the literature from 1998 through 2002. *Archives of Physical Medicine and Rehabilitation*.

[7] Cicerone K. D., Langenbahn D. M., Braden C. (2011). Evidence-based cognitive rehabilitation: updated review of the literature from 2003 through 2008. *Archives of Physical Medicine and Rehabilitation*.

[8] Turner-Stokes L., Pick A., Nair A., Disler P. B., Wade D. T., Cochrane Injuries Group (2015). Multi-disciplinary rehabilitation for acquired brain injury in adults of working age. *Cochrane Database of Systematic Reviews*.

[9] Koehler R., Wilhelm E., Shoulson I. (2012). *Cognitive rehabilitation therapy for traumatic brain injury: evaluating the evidence*.

[10] Chung C. S. Y., Pollock A., Campbell T., Durward B. R., Hagen S., Cochrane Stroke Group (2013). Cognitive rehabilitation for executive dysfunction in adults with stroke or other adult non-progressive acquired brain damage. *Cochrane Database of Systematic Reviews*.

[11] Cicerone K. D., Goldin Y., Ganci K. (2019). Evidence-based cognitive rehabilitation: systematic review of the literature from 2009 through 2014. *Archives of Physical Medicine and Rehabilitation*.

[12] Prigatano G. P., Wong J. L. (1999). Cognitive and affective improvement in brain dysfunctional patients who achieve inpatient rehabilitation goals. *Archives of Physical Medicine and Rehabilitation*.

[13] Wilson J. T. L., Pettigrew L. E. L., Teasdale G. M. (1998). Structured interviews for the Glasgow Outcome Scale and the Extended Glasgow Outcome Scale: guidelines for their use. *Journal of Neurotrauma*.

[14] Brehmer Y., Westerberg H., Bäckman L. (2012). Working-memory training in younger and older adults: training gains, transfer, and maintenance. *Frontiers in Human Neuroscience*.

[15] Constantinidou F., Thomas R. D., Scharp V. L., Laske K. M., Hammerly M. D., Guitonde S. (2005). Effects of categorization training in patients with TBI during post-acute rehabilitation: preliminary findings. *The Journal of Head Trauma Rehabilitation*.

[16] Wade S. L., Narad M. E., Shultz E. L. (2018). Technology-assisted rehabilitation interventions following pediatric brain injury. *Journal of Neurosurgical Sciences*.

